# Exploring the intersection between country context and priority setting to prevent maternal mortality: A multi-methods study comparing expected vs observed priorities in five countries to validate the obstetric transition model

**DOI:** 10.7189/jogh.13.04057

**Published:** 2023-06-09

**Authors:** Jewel Gausman, Dea Oviedo, Ana Langer, R Rima Jolivet

**Affiliations:** Department of Global Health & Population, Harvard T.H. Chan School of Public Health, Boston, Massachusetts, USA

## Abstract

**Background:**

The obstetric transition model suggests that, as countries economically develop, the primary causes of maternal mortality change. Countries are assigned to one of five stages based on their maternal mortality ratio to identify priorities for reducing maternal deaths based on predominant determinants of mortality at each stage. We aim to validate the obstetric transition model using data from six diverse low- and middle-income countries representing self-identified priorities for improving maternal health and measurement compiled in a multi-stakeholder process.

**Methods:**

We used multiple data sources from Bangladesh, Cote d’Ivoire, India, Mexico, Nigeria, and Pakistan, which included secondary data on country context and primary data derived from two sources: the content of multi-stakeholder meetings, called National Dialogues, which were organised around the 11 key themes identified in the World Health Organization’s “Strategies toward ending preventable maternal mortality” (EPMM) and follow-up key informant interviews conducted in five of the seven countries. We conducted the analysis in four phases examining, the country’s contextual profile, mapping the key themes and indicators to the model, exploring stakeholder prioritisation, and examining reasons for divergence from the model.

**Results:**

Our results suggest that the stages of the obstetric transition generally align with the social, epidemiological, and health systems characteristics that the model predicts to be associated with countries at each stage, with some deviation related to health system deficiencies and barriers to access. Stakeholder priorities in maternal health generally align with those predicted by the model. Equity and women’s rights emerged as a priority throughout all stages, not only within countries that are more advanced in the transition, as predicted by the model. Deviations between the model’s predictions and country-level prioritisation were often explained by context-specific challenges.

**Conclusions:**

This study is one of the first to validate the obstetric transition model using real data. Our findings support the validity of the obstetric transition model as a useful guide to aid decisionmakers in prioritising attention towards addressing maternal mortality. Country context, including equity, remains important to further inform priority-setting.

Despite notable declines in maternal mortality globally over the last several decades, few countries are on track to meet Sustainable Development Goal (SDG) target 3.1, which calls for a reduction in the global maternal mortality ratio (MMR) to less than 70 maternal deaths per 100 000 live births by 2030 [1]. The reductions in MMR observed globally have been inequitable, both within and across countries. Recent estimates suggest that the world’s poorest countries face an average MMR of 415 maternal deaths per 100 000 live births, 40 times higher than that observed in Europe and 60 times higher than that in Australia and New Zealand [2]. Within countries, the emphasis on reducing national averages in maternal mortality is thought to have caused countries to prioritise populations and conditions that were easiest to address rather than focus on equity [3]. To equitably reduce MMR, countries must be aware of the changing epidemiological and health systems landscape.

The obstetric transition model suggests that, as countries develop economically, both fertility and maternal mortality decline, and the primary causes of maternal mortality shift from predominantly direct causes to more indirect causes [4]. Direct maternal deaths are those “resulting from obstetric complications of the pregnant state (pregnancy, labour, and puerperium), and from interventions, omissions, incorrect treatment, or from a chain of events resulting from any of the above” [5]. Globally, haemorrhage, hypertensive disorders, obstructed labour, unsafe abortion, and sepsis are leading causes of direct maternal deaths. Indirect maternal deaths are those that result from previous existing disease, or disease that developed during pregnancy not due to direct causes, but due to being aggravated by pregnancy, such as those associated with existing cardiac or renal disease [5]. The obstetric transition model uses MMR to assign countries to one of five stages; however, the model includes some consideration of fertility, causes of maternal death, and parameters of quality of, access to, and utilisation of health services. The obstetric transition stages are summarised in **Box 1**. With shifting trends on the causes of maternal death, countries must also adapt strategies and interventions aimed at ending preventable maternal death.

Box 1 **Summary of the stages of the obstetric transition model [4].**

**Stage I**
− MMR greater than 1000 maternal deaths per 100 000 live births
− High fertility
− Majority of maternal deaths due to direct causes
− Majority of women do not have access to obstetric care or health services

**Stage II**
− MMR between 999-300 maternal deaths per 100 000 live births
− High fertility
− Majority of maternal deaths due to direct causes
− Increasing use of health care
− Other factors include lack of basic infrastructure, very low levels of women’s education, weak health systems, severe shortages of skilled birth attendants
− Poor quality of care contributes to limited demand for health services

**Stage III**
− MMR between 299-50 maternal deaths per 100 000 live births
− Fertility can be variable
− Direct causes of maternal death continue to be the most common.
− Access to and utilisation of health services is much higher than in previous stages, though access continues to be a barrier for a majority of the population.
− Quality of care becomes a major determinant of health outcomes as more pregnant women reach health facilities
− Facilities may be overloaded

**Stage IV**
− MMR less than 50 maternal deaths per 100 000 live births
− Low fertility
− Indirect causes, particularly noncommunicable diseases, become more dominant causes of maternal mortality
− Over-medicalisation may become an issue

**Stage V**
− MMR is lower than 5 maternal deaths per 100 000 live births; all avoidable maternal deaths are avoided
− Very low fertility
− Indirect obstetric causes are the main cause of maternal mortality
− Largely aspirational stage

For both direct and indirect maternal deaths, the underlying causes may be proximate or distal, but priorities change as countries move through obstetric transition stages. The obstetric transition model has previously been proposed as a framework which may suggest strategies for countries to follow to reduce MMR [3,6]. According to the model, countries with high MMRs should theoretically prioritise improvements in coverage of services (proximate) and access to health care and quality improvement (more distal) by increasing health system capacity to deliver basic life-saving interventions, addressing infrastructural challenges, and supporting demand generation by expanding women’s education. Countries with very low MMRs should theoretically find that reducing over-medicalisation in the health system (proximate), prevention of non-communicable diseases, and addressing issues of health equity among vulnerable and disadvantaged subpopulations (more distal), are the priority challenges to be addressed to make continued progress.

Distal determinants of maternal health and survival related to social, structural, political, and economic factors affect both direct and indirect causes of maternal mortality. In the early stages of the obstetric transition, distal determinants including structural, economic, and political factors are critical to strengthening the basic health system building blocks where there is an insufficient number of hospitals, a lack of financial resources allocated to health, and weak health system governance. At more advanced stages of the model, once again, upstream social and economic determinants take on greater significance because of the so-called excesses of wealth: increased non-communicable diseases related to obesity and sedentary lifestyle, overuse of interventions due to social and perverse economic incentives, etc. Thus, distal determinants play a role in ending preventable maternal deaths at both ends of the obstetric transition spectrum.

In 2015, the World Health Organization (WHO) released a report entitled “Strategies toward ending preventable maternal mortality” (EPMM), which outlined global targets and strategies for reducing maternal mortality in the 2015-2030 SDGs era [3], with a special focus on human rights and health system performance to eliminate disparities in access, quality, and outcomes of maternal care both within and between countries. This report established the MMR target for the SDG period: a global average MMR of fewer than 70 maternal deaths per 100 000 live births by 2030. To achieve this global goal, it further articulated tiered national targets for maternal mortality reduction based on each country’s baseline MMR in 2015. Countries with the highest baseline MMR (>420) should reduce it to at least 140 (no more than twice the global average), countries with baseline MMR under 420 should reduce it by at least two-thirds, and countries with low baseline MMR should achieve subnational equity for vulnerable populations. To reach these targets, the EPMM strategies highlight 11 EPMM key themes grounded in fundamental human rights principles of equity, non-discrimination, transparency, participation, and accountability. Together, they represent the full, broad spectrum of determinants of maternal health and survival, including social/structural, political, economic, and health system-level determinants. A comprehensive monitoring framework was developed with specific indicators associated with each key theme, to track national and global progress in improving maternal health [7,8].

To help countries in planning, tracking and accelerating progress toward ending preventable maternal mortality, the Women & Health Initiative of the Harvard T. H. Chan School of Public Health held a series of seven multi-stakeholder consultations between 2018 and 2020, known as National Dialogues, to understand national priority areas for improving maternal health and to support the adoption and use of EPMM indicators in national-level monitoring frameworks and other context specific advocacy efforts to drive improvement in those self-identified priority areas. The National Dialogues took place in seven countries: Bangladesh (February 2019), Cote d’Ivoire (November 2018), India (April 2019), Kenya (July 2018), Mexico (July 2019), Nigeria (March 2020), and Pakistan (October 2019), with each facing a different array of challenges for addressing the proximate and distal drivers of maternal mortality.

We aimed to validate the obstetric transition model using data derived during and after the National Dialogues on how countries prioritise the EPMM key themes and indicators based on their stage in the obstetric transition by exploring the degree of convergence between the recommended areas for intervention suggested by the obstetric transition model and the priorities identified by national-level stakeholders in each context.

## METHODS

**Figure 1:**
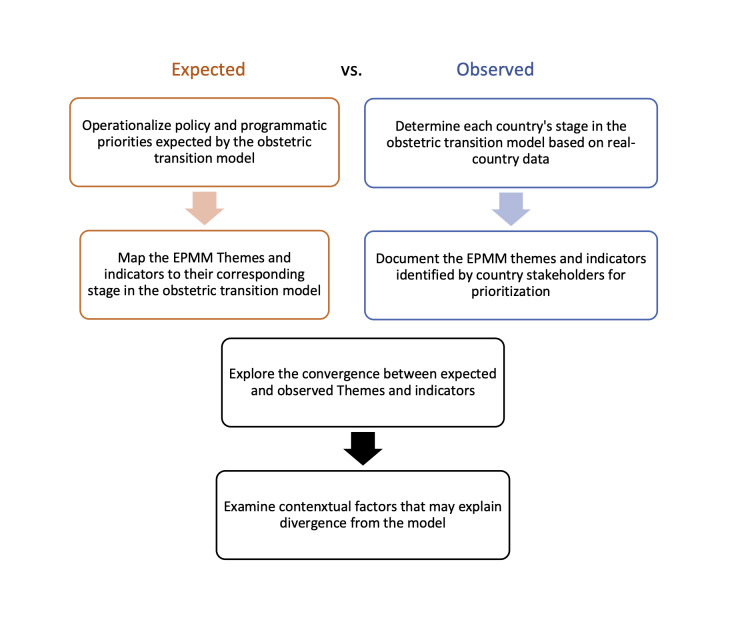
Study approach of comparing country priorities expected by the obstetric transition model to those observed.

To validate the obstetric transition model, we followed a multi-step process to first use theory and data to determine areas of focus that we would expect to observe based on the obstetric transition model which we then compared to the observed priorities in each country as identified by the selection of key themes and indicators by country stakeholders (**Figure 1**).

The four main objectives of our study were as follows:

1. Map the EPMM Themes and indicators to the priorities expected according to the obstetric transition model for countries at each stage of the model. This step forms the theoretical basis for how we determine what country-level priorities to expect based on the obstetric transition model.
2. Determine the stage in the obstetric transition model for each of the seven countries per the national MMR, and document the social, epidemiological, and health systems characteristics of the countries at each stage.
3. Explore how well the expected themes and indicators for countries at different stages of the obstetric transition correlate to real national priorities identified by multistakeholder groups in countries at those stages, and;
4. Examine contextual factors that might explain any convergence or divergence observed, including current events, challenges, concerns, or other interests that may have influenced the observed prioritisation of themes and indicators.

We follow the Consolidated criteria for reporting qualitative research (COREQ) guidelines [9]. The 32 item COREQ checklist can be found in Supplemental Table 1 in the **Online Supplementary Document**.

### Data sources

Second, we compiled textual data from key documents, including published literature and reports relating to the EPMM key themes [3], the obstetric transition model [4], and the National Dialogue meeting reports reflecting the consensus of all meeting participants [10]. Each of the National Dialogues included approximately 40-50 stakeholders representing expertise and commitment in the areas of each of the 11 EPMM key themes, including representatives from the Ministry of Health, United Nations and donor agencies, development partners, civil society advocates, and others from outside the health sector. We omitted Kenya from this analysis due to important process-related differences in how participants in the national dialogue prioritised themes and indicators from what was done in the other six countries.

We used both primary and secondary data sources. First, we extracted secondary data related to each country’s social and epidemiological profile relevant to maternal health and survival. Variables included those related to key health outcomes (MMR, fertility rate, the most common causes of maternal mortality, and modern contraceptive prevalence rate), health system indicators (percentage of births attendance by a skilled birth attendant, density of skilled health professionals per 10 000 population, percentage of births by caesarean section, percentage of deliveries in a health facility, out of pocket expenditure as a percentage of total expenditure on health), and sociodemographic and economic indicators, especially those indicative of women’s status (Gini coefficient, Human Development Index, Gender Inequality Index, percentage of population using improved sanitation facilities, primary school enrolment gender parity index, literacy rates for adult women) suggested as important by the model. Data sources included Countdown 2030, UNICEF Data Warehouse, Population Reference Bureau World Data Sheet, Institute for Health Metrics and Evaluation, World Health Organization Global Health Observatory, World Bank Open Data, United National Human Development Reports, and United Nations Statistical Annex.

Third, we collected primary data through key informant interviews (KIIs) conducted with participants from the National Dialogues to explore the influences that informed the prioritisation of each EPMM key theme and indicator. Candidates for these encounters were selected purposively based on their expertise or roles in the National Dialogue to include an array of perspectives; the final recruitment was a convenience sample based on availability.

Due to competing demands related to COVID-19 at the time of this research, we were able to include in primary data collection participants from five of the seven countries in which National Dialogues were held, representing at least one country in each geographical region and in each stage of the obstetric transition. The participants who provided KIIs were from Bangladesh (n = 3), India (n = 3), Mexico (n = 4), Nigeria (n = 2), and Pakistan (n = 3). KIIs ranged from 1-1.5 hours in duration. One of the authors (RRJ), who was trained in qualitative data collection and involved in facilitation and coordination of the National Dialogues conducted the KIIs. We prepared a semi-structured interview guide, developed based on the recommendations of key stakeholders in each country. Participants first reviewed the key themes and indicators that were prioritised by their country during the National Dialogue, as identified in their country’s final report. We then asked participants to reflect on major influences that they believed played a role in decisions around prioritisation of specific themes and indicators, and other contextual influences, such as donor priorities, political issues, or other external factors. KIIs were conducted via Zoom, and subsequently recorded and transcribed. Another author (JG) was present during the KIIs and took field notes. Two of the KIIs conducted in Mexico were conducted in Spanish and translated to English for analysis. Study team members performed quality checks to ensure accuracy of transcription.

### Analytical approach

To accomplish Objective 1, we used MMR to determine a country’s stage in the obstetric transition model, as specified in the obstetric transition model. Stage I is defined as MMR>1000 maternal deaths per 100 000 live births, stage II as between 300-999 maternal deaths per 100 000 live births, stage III as between 50-299 maternal deaths per 100 000 live births, stage IV as <50 maternal deaths per 100 000 live births, and stage V as <5 maternal deaths per 100 000 live births. We then examined the social and epidemiological profiles for each of the seven countries in relation to the other common factors at each stage presented in **Box 1**.

For Objective 2, we applied the obstetric transition model to identify those EPMM themes and indicators that are most relevant to countries at different stages in the obstetric transition. To do this, we identified and operationalised the recommended priority areas of focus identified by Souza et al. [4] at each stage of the obstetric transition. Using these recommended priority areas, we mapped the EPMM Themes and indicators to each obstetric transition phase using the priorities that emerged for each stage. Because the stages of the obstetric transition model overlap along a continuum of development in which the emphasis and/or the burden associated with factors shifts, some EPMM key themes or indicators may be relevant across more than one stage, or bimodally at each end of the development continuum.

For Objective 3, we compared the EPMM key themes and indicators prioritised by consensus during the multi-stakeholder dialogue in each country to the priorities we expected to observe based on our application of the obstetric transition model to the EPMM key themes and indicators. We used directed content analysis to analyse textual data extracted from the National Dialogue Reports [11], which uses a priori theory as an initial framework to code and interpret data. In applying this approach, we used deductive coding based on the EPMM key themes and indicators as our theoretical framework to identify which were prioritised and what commitments were made by stakeholders in relation to the identified priorities.

We then triangulated the results from Objectives 1, 2, and 3 to examine each country’s priorities in relation to their social and epidemiological profile to explore any contextual drivers that may have had an influence on prioritisation.

Last, for Objective 4, to provide an in-depth exploration of the role of country context in influencing the selection of EPMM key themes and/or indicators by country-level stakeholders, we conducted a thematic analysis of KII data to better understand how predominant context-specific factors or current events may have influenced priority setting and decision making in relation to concordance or discordance with expected priorities [12,13]. Data were analysed using a hybrid approach using both deductive and inductive coding [14]. First data were coded deductively with a priori codes identified according to whether the themes and/or indicators fit into the following categories:

1. Expected by the model and prioritised by stakeholders
2. Expected by the model and not prioritised by stakeholders
3. Not expected by the model but prioritised by stakeholders
4. Neither expected by the model nor prioritised by stakeholders

Once this classification was complete, we used inductive coding within each category to explore specific areas of convergence and divergence between the model and real-world experience. KIIs were recoded as new codes emerged. Two coders coded each KII and revised the codes until consensus was reached. We used Dedoose version 9.0.17 for the qualitative analysis [15].

### Ethical approval

The Harvard T.H. Chan School of Public Health’s Institutional Review Board approved this study. All participants provided verbal informed consent to participate.

## RESULTS

### Objective 1

Countries are presented according to their stage in the obstetric transition model (**Table 1**), defined by MMR. As predicted by the model, countries in the early obstetric transition stages had higher fertility and lower modern contraceptive prevalence rates (mCPR) than those in later ones. Pakistan is a notable exception, with a mCPR of only 23%. Mexico in stage IV (22.4%) and Bangladesh in stage III (33.1%) had the highest percentages of maternal deaths due to indirect causes.

**Table 1:** Social, epidemiological, and health systems profiles according to stage in the obstetric transition

Stage in obstetric transition	Stage 2		Stage 3			Stage 4	Data Source
**Country**	**Côte d’Ivoire**	**Nigeria**	**Bangladesh**	**India**	**Pakistan**	**Mexico**	
**Maternal and reproductive health status**							
MMR	617	917	173	145	140	33	Countdown 2030 [16]
Fertility rate	4.6	5.3	2.3	2.2	3.6	2.1	UNICEF Data Warehouse [17]
Top causes of maternal mortality							
*Indirect maternal deaths*	11%	5.1%	31.1%	18%	8.4%	22.4%	Institute for Health Metrics and Evaluation [18], Population Reference Bureau [19]
*Maternal haemorrhage*	14.7%	28.3%	32.0%	36.0%	24.8%	16.9%	
*Maternal hypertensive disorders*	10.6%	8.7%	10.0%	8.5%	37.0%	20.1%	
*Sepsis and infection*	6.6%	4.7%	0.2%	4.6%	5.0%	3.3%	
*Abortion and miscarriage*	19.3%	14.4%	2.3%	3.2%	2.9%	4.5%	
*Maternal deaths aggravated by HIV*	2.2%	1.9%	0.0%	0.1%	0.0%	10.0%	
*Other direct causes*	26.9%	24.1%	11.2%	25.5%	15.2%	16.8%	
*Late maternal deaths*	8.6%	12.8%	13.2%	4.7%	6.5%	10.1%	
mCPR	20%	12%	59.0%	48%	23%	70%	
**Access to care**							
Percentage of births attended by a SBA	73.6%	43.3%	52.7%	81.4%	69.3%	96.4%	UNICEF Data Warehouse [17]
Density of skilled health professionals per 10 000 population							
*Doctors*	2.3	3.8	5.8	8.5	9.8	23.8	World Health Organization Global Health Observatory [20]
*Nurses*	6	11.8	4.1	17.3	6.7	24	
Percentage of births by caesarean section	3.3%	2.7%	30.7%	17.2%	14.1%	40.7%	Countdown 2030 [16]
Percentage of deliveries in a health facility for women aged between 15-49	70%	39%	53%	79%	66%	97%	UNICEF Data Warehouse [17]
**Health systems and financing**							
Out of pocket expenditure as a percentage of total expenditure on health	39.4%	76.6%	73.9%	62.7%	56.2%	42.1%	World Bank Open Data [21]
**Sociodemographic and economic development**							
Gini coefficient (0 = complete equity)	41.5	35.1	32.4	37.8	33.5	45.4	World Bank Open Data [21]
Human development index (0 = less developed)	0.538	0.601	0.632	0.645	0.557	0.779	UNDP Data Center [22]
Gender inequality index (0 = gender equality)	0.638	-	0.537	0.488	0.538	0.322	UNDP Data Center [22]
Percentage of population with access to an improved water source	72%	58%	97%	93%	91%	99%	World Bank Open Data [21]
Percentage of population using improved sanitation facilities	32%	39%	48%	59%	59%	91%	World Bank Open Data [21]
School enrolment, primary (gross), GPI	.94	.94	1.07	1.15	.859	1.00	World Bank Open Data [21]
Literacy rates for adult females (aged ≥15)	40%	53%	71%	66%	46%	95%	World Bank Open Data [21]

MMR – maternal mortality ratio, SBA – skilled birth attendant, HIV – human immunodeficiency virus, mCPR – modern contraceptive prevalence rate, GPI – gender parity index

Metrics related to health care access generally suggested that a larger percentage of the population has access at later stages of the obstetric transition, with Cote d’Ivoire being a notable exception. In Cote d’Ivoire, 73% of births are attended by a skilled birth attendant and 70% of births occur in health facilities, which is considerably higher than in Nigeria in stage II and Bangladesh and Pakistan in stage III; however, its density of skilled health professionals remained comparatively low. In Cote d’Ivoire, there are 2.3 doctors and 6 nurses per 10 000 population compared to 3.8 doctors and 11.8 nurses per 10 000 population in Nigeria, and 8.5 doctors and 17.3 nurses per 10 000 population in Pakistan. Countries in stage II have very few births delivered by caesarean section, with an increasing percentage as countries advance through later stages of the obstetric transition. The percentage of births by caesarean section for all countries in stage III (ranging from 14.1% in Pakistan to 30.7% in Bangladesh) is substantially higher than among countries in stage II. Similarly, only 2.7% of births are by caesarean section in Nigeria in stage II compared to Mexico in stage IV, which has the highest number of births (40.7%) of all countries.

Indicators related to economic and human development suggest that countries in the more advanced phases of the obstetric transition tend to have a larger percentage of the population with access to improved drinking water and sanitation and higher levels of female literacy. Further, countries in more advanced stages of the obstetric transition also scored higher on the Human Development Index and have higher gender equality when compared to those in earlier stages. However, Pakistan is a notable exception, with lower literacy rates among women (46%) than the other two countries in stage III and the lowest gender parity in primary school enrolment out of any of the countries included in the study (0.859).

### Objective 2

Supplemental Table 1 in the **Online Supplementary Document** maps the 11 EPMM key themes and indicators to stages II, III, and IV of the obstetric transition model and provides the rationale for assigning indicators to specific stages based on the programmatic priorities suggested by the model.

The obstetric transition model posits that countries in stage II would benefit from a focus on developing the basic physical infrastructure and human resources to deliver essential maternal health services. Thus, we assigned EPMM key themes and indicators that focus on ensuring geographic distribution of emergency obstetric care (themes 10 and 4), universal coverage of essential health services (theme 8), and authorising midwives to deliver emergency services (theme 3) to this stage. Similarly, we assigned themes focused on ensuring adequate allocation of financial resources to support basic health infrastructure and out of pocket expenditures (theme 6), and ensuring that financial, legal, and regulatory frameworks are in place to support the provision of basic health services (theme 3) to stage II. In stage II, we also included themes that focus on empowering women and girls and human rights (theme 1 and theme 4), and ensuring facilities are responsive to the specific needs of women and girls (theme 10) as we considered them to be closely related to demand generation.

Some themes are relevant to both stage II and stage III, as the model expects countries to continue to focus on primary prevention and access as needed as countries transition between stages. However, stage III begins to emphasise issues related to quality of care, such as improving skilled birth attendance and management of complications, addressing facility-level issues, such as timeliness of care, staffing, etc. and focusing on secondary and tertiary prevention. This shift in focus is illustrated by the emergence of themes 2, 7, and 11 in stage III, which focus on integrating maternal and newborn care (theme 2), improving equity in care (theme 7), and improving accountability (theme 11) as important. Changes in the specific indicators expected under each theme help to differentiate the stages. For example, theme 5 (improve metrics, measurement, and data) is thought to be important to both stage II and stage III, but the focus in indicators shifts from a focus on coverage (Indicator 5.1: “presence of a national set of indicators with targets and annual reports to inform annual health sector reviews and other planning cycles”) in stage II to a focus on higher level prevention efforts and quality concerns (Indicator 5.2: “maternal death review coverage”) in stage III.

In stage IV, the model’s focus shifts from addressing direct causes of maternal mortality and improving basic quality of care to an emphasis on indirect causes, addressing more advanced quality of care issues, eliminating delays within the health system, reducing overmedicalisation, and addressing subpopulation inequities. Ensuring accountability to improve quality of care and equity (theme 11) is aligned with many of these priority areas; however, the most pronounced shift in emphasis occurs regarding addressing issues of equity. While there is overlap in the rights-based EPMM key themes that are priority areas in stage II, the associated indicators that align with stage IV focus more directly on issues of equity. For example, the expected indicators for theme 1 (empowering women, girls, and communities) shift from those that relate primarily to ensuring women are able to access to health services (Indicators 1.1: “presence of laws and regulations that guarantee women’s access to SRH care and information” and 1.2: “gender parity index”) to a focus on non-discrimination (Indicator 1.3: “non-discrimination on the basis of sex”). Similarly, in theme II, which focuses on integrating maternal and newborn health, Indicators 2.2: “maternity protection in accordance with ILO Convention 183” and 2.3: “international code of marketing of breastmilk substitutes” emerge as important through an equity lens. Last, while Theme 7 (address inequities in access to and quality of sexual, reproductive, maternal, and newborn health care) focuses specifically on addressing inequities in access to and quality of services, a specific emphasis on Indicator 7.2: “equity stratifiers to address subpopulation inequities related to wealth, educational level, geography, and age”, emerges.

### Objectives 3 and 4

For countries in all stages, the prioritised themes and indicators aligned well with those that were expected based on each country’s stage in the obstetric transition (Supplementary Tables 3 and 4 in the **Online Supplementary Document**).

#### Countries in stage II

For countries in stage II (Cote d’Ivoire and Nigeria), 83.3% (five out of six) of the prioritised themes were among those that were expected based on the countries’ stage in the obstetric transition.

Cote d’Ivoire prioritised themes 5 (improve metrics, measurement systems, and data quality) and 10 (strengthen health systems to respond to the needs and priorities of women), as was expected; however, stakeholders also prioritised theme 2 (integrate maternal and newborn health, protect and support the mother-baby dyad) which is an expected priority for countries in stage III and later. All the indicators prioritised by Cote d’Ivoire for the two expected themes were among those predicted by the model based on the country’s stage, except for Indicator 5.2: “maternal death review coverage”, which was expected for countries in stage III. Per the secondary data on country context (**Table 1**), Cote d’Ivoire has a substantially higher percentage of births attended by a skilled birth attendant and births occurring in health facilities. In Cote d’Ivoire, 70% of births occur in facilities, which is substantially higher than the 39% that occur in facilities in Nigeria. Cote d’Ivoire’s institutional birth rate is also higher than that in Bangladesh (53%) and in Pakistan (66%) in stage III of the model. Despite the relatively high percentage of institutional deliveries for its stage, the percentage of births by caesarean section in Cote d’Ivoire is 3.3%, similar to that observed in Nigeria (2.7%).

For themes 3 (ensure country ownership, leadership) and 4 (apply a human rights framework), Nigeria prioritised only indicators that were expected; however, for theme 6 (allocate adequate resources and effective health care financing), one of the two indicators prioritised was predicted by the model, while the other was not. Indicator 6.1: “percent of total health expenditure spent on RMNCH” was among those expected for countries in stage II, but Indicator 6.3, “annual review of health sector spending”, was not; however, qualitative data supports this selection. In the National Dialogue, participants cited concern over the lack of investment in reproductive and maternal health, coupled with a lack of transparency and accountability in budgeting, which they believed resulted in poor-quality maternity services. Poor quality was then thought to affect demand for services negatively. A similar sentiment was also echoed in KIIs. Several participants expressed frustration by the lack of government investment in maternal health care, cited a need for more accountability and transparency in financing, and described how both issues manifested in poor quality of care. A quote from a participant from Nigeria captures this sentiment:

So, it's about the allocation to health... if we go back to the Abuja Declaration of 2001, that says that countries should devote at least 10% of the annual budget to health… Nigeria is still very low, below five [percent at the federal level]...We have some states that are performing very poorly...we need to allocate resources appropriately to the burden of disease, we need to be able to allocate this investment appropriately to the level of care...nothing [is] stopping us from having functional clinics and functional hospitals...[they] should be full-fledged to actually help deliver on the five components of primary care like we see in a developed countries, so for us to do all these things, the funds are actually required... Are [we] really investing [our funds] appropriately... or we’re just investing these resources or allocating them to frivolous things? *– Key informant, Nigeria*

#### Countries in stage III

For countries in stage III (Bangladesh, India, and Pakistan), 77.8% (seven out of nine) of the themes prioritised were the expected ones. In India, all three of the prioritised Themes and associated indicators were among those expected for countries in stage III. While also in stage III, Bangladesh and Pakistan each prioritised one theme that was expected for countries in stage II. Among those expected for countries in stage II, Bangladesh prioritised theme 3 (ensure country ownership, leadership) and Pakistan prioritised theme 4 (apply a human rights framework).

The indicators selected for the themes prioritised by India all relate to improving access to health services through coverage, health worker density, availability, and service readiness, as was expected for countries continuing to work on expanding basic access and improving quality of care in stage III. During the National Dialogue, participants in India described ongoing efforts to develop national guidelines to establish and strengthen midwifery practice, antenatal care, postnatal care, and care for high-risk pregnancies, coupled with strong interest in addressing issues related to the social determinants of health and advancing equity. The focus on expanding access to and coverage of quality services was described as a critical component of improving health equity; however, an emphasis on multi-sectoral engagement, including strengthening linkages between the public and private sectors were thought to be essential as part of a focus on addressing social determinants. This relationship was illustrated in a discussion between two participants:

It's a triangle, the government wanted to provide services. They invited women to their facilities. Women experienced inequities and they had difficulties and there were a lot of gaps...And because of the situation, actually it's a macro issue. There was a policy which did not look at the inadequacies and before fulfilling those gaps, they started inviting women. As a result, women had negative experiences, which led to this whole need for strengthening services before inviting women to the facilities...I would also look at it as a gender issue. Because we have gender discrimination, we do not value women and their lives, and we assume that you can give anything of any quality to women who come to your place. So, the larger issue is again gender mindset in the health system...But yes, it's a circle or a triangle, where the policy implementation and again policy change. – Key Informant A, India

When we made [women] aware that these are your rights: you should have a clean bed, you should have a person talking to you to give you information... and what quality care [is], then, they started demanding [it]. And because of that, it came out that strengthening health systems and workforce is required... [because] once [women] reach [health facilities to deliver]... I have myself seen that [there are sometimes] 10 mothers in two beds, [with] one health care provider ...What are the expectations from healthcare providers? Respectful Maternity Care has come into the syllabus now of nursing and midwifery. I think it is going to make a difference. *–Key informant B, India*

While thought to be more relevant for countries in stage II, Bangladesh’s prioritisation of theme 3 (prioritise country ownership, leadership and supportive legal, regulatory and financial

mechanisms), and the associated Indicators 3.1, “costed implementation plan for MNCH” and 3.2, “midwives are authorised to deliver basic emergency obstetric and newborn care (EmONC)” relate to developing basic health system infrastructure and reinforcing primary prevention efforts by ensuring access to essential health services. The country’s epidemiological profile (**Table 1**) points toward weaknesses in basic service delivery infrastructure. When compared to the other countries in stage III, the percent of births attended by a skilled birth attendant, density of skilled health professionals per 10 000 population, and the percentage of deliveries in a health facility are lower than the other two countries in stage III. In Bangladesh, for example, 43.3% of deliveries are attended by a skilled birth attendant, compared to 52.7% in India and 81.4% in Pakistan.

Key informants also emphasised challenges facing Bangladesh associated with basic service delivery for maternal health services, citing a nascent midwifery program that has been implemented to expand access to services, and shortcomings related to availability of EmONC, thereby reinforcing the rationale for the selection of Theme 3 and the indicators that were prioritised. As one participant said,

We have the initiative for introduction of midwifery system in our context, that is a very new and recent program and work is [under]way for working on different aspects from planning the required number of midwives, then development of training curriculum for the midwives then implementation of the training program...and developing job responsibilities for midwife. Then posting of the midwife in different places, you know all these things are going on but, but there are also challenges as well...Bangladesh [was] one of the pioneer countr[ies] in implementation of EmONC ...we started this process in mid-1990s. But we still could not become this country of excellence in demonstrating the functionality of the facilities in terms of availability...of all the signal functions for EmONC... *– Key informant, Bangladesh*

Theme 5 (improve metrics, measurement systems, and data quality) was among those expected for countries in stage III. Stakeholders in Bangladesh then prioritised Indicator 5.2, “maternal death review coverage”, which is associated with countries in stage III; however, they also selected Indicator 5.1, “presence of a national set of indicators with targets and annual reports to inform annual health sector reviews and other planning cycles”, which was not (the latter was again associated with development of basic health infrastructure, thought to be more important for countries in stage II). In discussing the reasons for this prioritisation, one participant emphasised the need for a stronger set of indicators to be used in monitoring efforts, but also better coordination in the use of the indicators,

All the stakeholders, plus NGOs who are involved in safe motherhood... once the indicators are selected, they could make it as part of their indicators of their projects. So the government [could] see that... these projects are getting these indicators right...all these things are already there, but measurement has to be more... more acute, more accurate and more up to date. *– Key informant, Bangladesh*

Pakistan’s prioritisation of theme 4 (apply a human rights framework) was accompanied by their selection of Indicators 4.1, “proportion of women aged 15-49 who make their own informed decisions regarding sexual relations, contraceptive use, and reproductive health care” and 4.3, “geographic distribution of facilities that provide basic and comprehensive EmoNC”. Theme 4, along with both indicators selected, were expected for countries in stage II. Pakistan’s contextual data (**Table 1**) shows that the country lags behind the other countries in stage III regarding indicators indicative of the status of women, including modern contraceptive use, women’s literacy rates and gender parity in school enrolment. Notably, only 23% of women in Pakistan use modern contraception, compared to 48% in India and 59% in Bangladesh. Similarly, only 46% of women are literate in Pakistan, compared to 66% in India and 71% in Bangladesh. In terms of geographic access to maternal health services, national-level data show that overall, nearly 70% of births are attended by a skilled birth attendant, which is higher than Bangladesh, but lower than India. Key informants, however, emphasised the importance of addressing subnational access disparities in the country. As indicated by one participant,

Especially where MMR is high, for example, in Sindh, and also in Khyber Pakhtunkhwa... also in Balochistan... [these areas] really need support to strengthen the efforts to bring down the MMR and also enhance the access to services... *– Key informant, Pakistan*

While theme 6 (allocate adequate resources and effective health care financing) was among the Themes expected for countries in stage III, Pakistan selected all three indicators associated with Theme 6, of which only Indicator 6.2, “out of pocket expenditure as a percentage of total expenditure on health” is expected for countries in stage III. Indicator 6.1 (“the per cent of total health expenditure spent on reproductive, maternal, newborn, and child health”) is expected for countries in stage II as it is thought to be associated with developing basic health infrastructure. Participants in the Pakistan National Dialogue emphasised the importance of accountability in resource allocation as being a fundamental component of ensuring implementation of quality services. Key informants reinforced this sentiment, with one participant stating,

And so what I see is non-availability of contraceptives, because of the funds are withdrawn. Now, even though there's commitments...the government votes at the federal and on the provincial level, but [is] not able to fulfill their resource allocations [and] commitments made in the last year and currently this year. I think one of the areas that were discussed in one of the forums that I was bought on was a political commitment. When we talk about, again, financial matters. Again, there's a divide between what to commit and what's available, made available for expenditure. So that big gap between allocation expenditure reflects weaknesses in political commitment. *– Key informant, Pakistan*

#### Countries in stage IV

Mexico was the only country in stage IV included in the study. Unlike the other countries, Mexico prioritised four key themes, as participants in the National Dialogue could not agree on the elimination of any one of the four. Half (two out of four) of the Themes prioritised in Mexico were among those that were expected. Themes 4 (apply a human rights framework) and 7 (address inequities in access to and quality of sexual, reproductive, maternal, and newborn health care) were two themes expected for countries in stage IV, but interestingly, stakeholders also prioritised themes 6 (allocate adequate resources and effective health care financing) and 10 (strengthen health systems to respond to the needs and priorities of women), both of which were expected to be prioritised by countries in stage III.

The indicators selected by stakeholders in Mexico for both themes that were expected to be prioritised by countries in stage IV, and those that were not focused on equity. A focus on vulnerable populations, such as indigenous groups and ethnic minorities, adolescents, and rural-urban migrants, was expected among countries in stage IV, and was reflected as a priority in KIIs. However, Indicator 4.1, “proportion of women aged 15-49 who make their own informed decisions regarding sexual relations, contraceptive use, and reproductive health care” relates to issues of women’s rights and status, and was expected to be prioritised by countries in stage II. Even though the contextual data for Mexico indicates that a large proportion of women use modern contraception (70%), most women are literate (95%), and that the county has achieved gender parity in school enrolment, participants emphasised that the country still faces challenges in ensuring women are able to make their own informed decisions relating to their sexual and reproductive health care. One participant stated,

I think that the very health system does not allow women to make their own choices – and obviously that has repercussions along the entire chain of the continuum of care. *– Key informant, Mexico*

Another participant emphasised that the health system takes advantage of women who do not understand their rights,

From the culture, the normalization of the violation of rights, the invisibility of these abuses and a lack of exercising one’s rights. So there are women who do not exercise their rights because they do not know what these rights are. – *Key informant, Mexico*

Mexico also emphasised the continued need to focus on quality of care by prioritising Indicator 10.3, (“percentage of facilities that demonstrate facility readiness to deliver specific services, i.e. family planning”), despite it being associated with earlier stages of the obstetric transition. Participants in the National Dialogue described a need to ensure that primary health facilities have the capacity to handle normal deliveries, and to strengthen the integration of family planning services into maternal health services, as it is often considered to be a standalone intervention unrelated to maternal mortality. Participants in Mexico discussed that the transition to lower maternal mortality allowed for a deeper emphasis on improving quality of care, including increasing contributions of professional midwives, legal protections, and reducing obstetric violence.

## DISCUSSION

Our results suggest that the stages of the obstetric transition, as defined by MMR, generally align with the social, epidemiological, and health systems characteristics that the model predicts to be associated with countries at each stage. Further, we find that stakeholder priorities in maternal health are generally aligned with those predicted by the model. In our study, deviations between what the model predicts and what is reflected through country-level prioritisation are often explained by specific issues that relate to challenges within a country’s context.

Our assignment of countries to stages is similar to previous research that used data from 29 countries obtained from the WHO’s Multi-Country Survey on Maternal and Newborn Health 2010-2012 to stage countries according to the model. The study concluded that the model is a valid framework for developing strategies to reduce maternal mortality according to a country's stage in the obstetric transition [6]. We expand these results further by exploring how closely real experiences of national-level prioritisation correspond to priorities expected based on a country’s stage in the obstetric transition model, and where important differences exist.

While our results generally support the validity of the model as a tool to aid in the development of strategies for advancing maternal mortality reduction, they also emphasise the importance of country context in identifying strategies beyond those expected by the model. For example, in Cote d’Ivoire, national data point to high coverage of maternal health services but low quality of care, prompting the country to prioritise strategies more consistent with stage III; however, the country’s MMR remains at a level consistent with stage II. Bangladesh and Pakistan highlight another example. Both countries are in stage III of the obstetric transition; however, they both have considerably lower health worker density and coverage of skilled birth attendance and institutional deliveries compared to what would be expected at stage III, and what is seen for example in India. Thus, in those contexts, a continued focus on stage II priorities, such as strengthening basic access and demand generation appears to be warranted. An area not considered explicitly by the model is the strengthening of health systems through improved resource allocation, financing, and accountability, which emerged as priorities for countries in all stages of the obstetric transition.

We also argue that countries applying the obstetric transition model to support development of strategies to address maternal mortality should be cognisant of health disparities, gender inequities, and the burden of maternal morbidity and mortality within vulnerable population groups at all stages of development, in keeping with universal human rights principles. While the model primarily focuses on health disparities at more advanced stages of the obstetric transition, we believe that equity is important to address at all stages of the obstetric transition and should not be overlooked in contexts of high maternal mortality.

Our study has several strengths and limitations to highlight. This study is one of the first to validate and apply the obstetric transition model using real country data [6]. We use an innovative design that incorporates numerous qualitative and quantitative data sources that reflect multiple dimensions of a country’s social, epidemiological, and health system’s profile, combined with key informant perspectives about real priority setting to improve maternal health. Simultaneously, while our data provide depth, our sample of both countries and key informants within countries is relatively small, which limits our study’s global generalisability. However, we found some evidence of saturation, as we found strong convergence and repetition of our themes evidenced within our study sample. Further, data collection occurred during the COVID-19 pandemic, which reduced stakeholder participation due to competing national priorities and emergency response. Finally, the National Dialogues occurred between 2018 and 2020, but our KIIs were not conducted until 2021. While participants generally remembered the content of the meetings, and the KII facilitator reminded participants of the themes and indicators prioritised in the National Dialogue at the beginning of the interview, participants may not have remembered the full content of the meeting, or the country context at the time of the National Dialogue, resulting in the possibility of some recall error.

## CONCLUSIONS

Our results support the validity of the obstetric transition model as a useful guide to support decisionmakers in selecting priorities to address maternal mortality. The model has been used globally by countries to monitor progress in reducing maternal mortality and to identify priorities for action [23-25]. Our results suggest that countries using the model also consider their own context, including considerations related to equity, as such issues remain important to further inform priority-setting that may transcend the model’s stages.

## Additional material


Online Supplementary Document
