# Supplementary material for: Exploring the intersection between country context and priority setting to prevent maternal mortality: A multi-methods study comparing expected vs observed priorities in five countries to validate the obstetric transition model
Source: J Glob Health. 2023 Jun 9;13:04057. doi: 10.7189/jogh.13.04057 (PMC10256276; doi:10.7189/jogh.13.04057)
Supplement: Online Supplementary Document [file jogh-13-04057-s001.pdf]

COREQ (COnsolidated criteria for REporting Qualitative research) Checklist

A checklist of items that should be included in reports of qualitative research. You must report the page number in your manuscript where you consider each of the items listed in this checklist. If you have not included this information, either revise your manuscript accordingly before submitting or note N/A.

Topic	Item No.	Guide Questions/Description	Reported on Page No.
**Domain 1: Research team and reflexivity**			
Personal characteristics			
Interviewer/facilitator	1	Which author/s conducted the interview or focus group?	6
Credentials	2	What were the researcher's credentials? E.g. PhD, MD	6
Occupation	3	What was their occupation at the time of the study?	6
Gender	4	Was the researcher male or female?	NA
Experience and training	5	What experience or training did the researcher have?	6
Relationship with participants			
Relationship established	6	Was a relationship established prior to study commencement?	6
Participant knowledge of the interviewer	7	What did the participants know about the researcher? e.g. personal goals, reasons for doing the research	6
Interviewer characteristics	8	What characteristics were reported about the inter viewer/facilitator? e.g. Bias, assumptions, reasons and interests in the research topic	6 and 16
**Domain 2: Study design**			
Theoretical framework			
Methodological orientation and Theory	9	What methodological orientation was stated to underpin the study? e.g. grounded theory, discourse analysis, ethnography, phenomenology, content analysis	7
Participant selection			
Sampling	10	How were participants selected? e.g. purposive, convenience, consecutive, snowball	6
Method of approach	11	How were participants approached? e.g. face-to-face, telephone, mail, email	6
Sample size	12	How many participants were in the study?	6
Non-participation	13	How many people refused to participate or dropped out? Reasons?	16
Setting			
Setting of data collection	14	Where was the data collected? e.g. home, clinic, workplace	6
Presence of non-participants	15	Was anyone else present besides the participants and researchers?	6
Description of sample	16	What are the important characteristics of the sample? e.g. demographic data, date	16
Data collection			
Interview guide	17	Were questions, prompts, guides provided by the authors? Was it pilot tested?	6
Repeat interviews	18	Were repeat inter views carried out? If yes, how many?	6
Audio/visual recording	19	Did the research use audio or visual recording to collect the data?	6
Field notes	20	Were field notes made during and/or after the inter view or focus group?	6
Duration	21	What was the duration of the inter views or focus group?	6
Data saturation	22	Was data saturation discussed?	16
Transcripts returned	23	Were transcripts returned to participants for comment and/or	NA

Topic	Item No.	Guide Questions/Description	Reported on Page No.
		correction?	
**Domain 3: analysis and findings**			
Data analysis			
Number of data coders	24	How many data coders coded the data?	6
Description of the coding tree	25	Did authors provide a description of the coding tree?	6
Derivation of themes	26	Were themes identified in advance or derived from the data?	6-7
Software	27	What software, if applicable, was used to manage the data?	7
Participant checking	28	Did participants provide feedback on the findings?	NA
Reporting			
Quotations presented	29	Were participant quotations presented to illustrate the themes/findings? Was each quotation identified? e.g. participant number	9-14
Data and findings consistent	30	Was there consistency between the data presented and the findings?	9-14
Clarity of major themes	31	Were major themes clearly presented in the findings?	9-14
Clarity of minor themes	32	Is there a description of diverse cases or discussion of minor themes?	9-14

Developed from: Tong A, Sainsbury P, Craig J. Consolidated criteria for reporting qualitative research (COREQ): a 32-item checklist for interviews and focus groups. *International Journal for Quality in Health Care*. 2007. Volume 19, Number 6: pp. 349 – 357

Once you have completed this checklist, please save a copy and upload it as part of your submission. DO NOT include this checklist as part of the main manuscript document. It must be uploaded as a separate file.

Supplementary Table 1: Mapping of the 11 EPMM themes and indicators to Stages 2, 3 and 4 of the obstetric transition model

Theme/Indicator	Stage of the Obstetric Transition<br>Stage 2	Stage 3	Stage 4
	1. Developing the basic infrastructure (including human resources)<br>2. Maternal-mortality primary prevention measures (e.g. access to family planning, safe abortion, iron supplementation, insecticide treated nets, and governance to remove barriers)<br>3. Management of direct causes of death<br>4. Demand generation to address first and second delay and increase access and utilization (going to facility is a reasonable alternative)	1. Elevating focus on secondary and tertiary prevention (but maintaining focus on primary prevention, as needed)<br>2. Access remains an issue in some settings<br>3. Skilled birth attendance and appropriate management of complications and disabilities (less about coverage and more about skill)<br>4. Intrahospital issues (3rd delay), lack of timely care, quality of care, staffing, etc.	1. Indirect causes of maternal mortality, particularly noncommunicable diseases<br>2. Addressing quality of care issues (more advanced)<br>3. Eliminating delays within health systems (referral delays, dysfunction, etc.)<br>4. Addressing issues of over-medicalization<br>5. Subpopulation inequities (gender inequality, structural violence, vulnerable populations)
**Theme 1: Empower women, girls, and communities**			
1.1 Presence of laws and regulations that guarantee women access to SRH care, info, and	4	2	
1.2 Gender Parity Index	4	2	
1.3 Non-discrimination on the basis of sex			5
**Theme 2: Integrate maternal and newborn health, protect and support the mother-baby**			
2.1 Presence of protocols/policies on combined care of mother and baby, immediate breastfeeding, and observations of care		1,4	
2.2 Maternity protection in accordance with ILO Convention 183			5
2.3 International code of marketing of breastmilk substitutes			5
**Theme 3: Ensure country ownership, leadership, and supportive legal, regulatory, and**			
3.1 Costed implementation plan for MNCH	1		
3.2 Midwives are authorized to delivery basic EmONC	1,3		
3.3 Legal status of abortion	2		
**Theme 4: Apply a human-rights framework to ensure that high-quality reproductive, maternal, and newborn health care is available, accessible, and acceptable to all who need it**			
4.1 Proportion of women aged 15-49 who make their own informed decisions regarding sexual relations, contraceptive use, and reproductive health care	4		
4.2 Costed implementation plan for MNCH	1		
4.3 Geographic distribution of facilites that provide basic and comprehensive EmOC	1,4		
**Theme 5: Improve metrics, measurement systems, and data quality to ensure that all**			
5.1 Presence of a national set of indicators with targets and annual report to inform annual health sector reviews and other planning cycles	1		
5.2 Maternal death review coverage		1,4	
**Theme 6: Allocate adequate resources and effective health care financing**			
6.1 Percentage of total health expenditure spent on RMNCH	1		
6.2 Out of pocket expenditure as a percentage of total expenditure on health	1,4	2	
6.3 Annual reviews are conducted of health spending from all financial sources included spending on RMNCH, as part of broader health sector reviews			
**Theme 7: Address inequities in access to and quality of sexual, reproductive, maternal, and newborn healthcare**			
7.1 Health worker density and distribution (per 1,000 population)		2	
7.2 Equity stratifiers			5
7.3 Presence of a national policy/strategy to ensure engagement of civil society organization		4	3,5
**Theme 8: Ensure universal health coverage for comprehensive sexual, reproductive, maternal, and newborn healthcare**			
8.1 Coverage of essential health services	1,4	2	
8.2 If fees exist for health services in the public sector, are women of reproductive age (15-49)	4	2	
**Theme 9: Address all causes of maternal mortality, reproductive and maternal morbidities, and related disabilities**			
9.1 Legal status of abortion	2	1	
9.2 Demand for family planning satisfied through modern methods of contraception	2	1	
**Theme 10: Strengthen health systems to respond to the needs and priorities of women**			
10.1 Availability of functional EmOC facilities	1,4	1,2,3,4	
10.2 Density of midwives, by district (by births)	1,4	2	
10.3 Percentage of facilities that demonstrate readiness to delivery specific services: family	1,2, 3	1,2,3,4	
**Theme 11: Ensure accountability in order to improve quality of care and equity**			
11.1 Civil registration coverage of cause of death (percentage)		1,3,4	1,2,3,5
11.2 Presence of a national policy/strategy to ensure engagement of civil society organization			1,2,3,5
11.3 Transparency stratifier			

Supplementary Table 2: Expected Versus Prioritized EPMM Key Themes By Country and Stage of Obstetric Transition

	**Stage 2**		**Stage 3**			**Stage 4**
**Key Theme**	Cote d'Ivoire	Nigeria	Bangladesh	India	Pakistan	Mexico
Theme 1						
Theme 2	X					
Theme 3		X	X			
Theme 4		X			X	X
Theme 5	X		X			
Theme 6		X			X	X
Theme 7				X		X
Theme 8				X		
Theme 9						
Theme 10	X		X	X	X	X
Theme 11						

Key

	Expected For Stage 2
	Expected for Stage 3
	Expected for Stage 4
X	Prioritized Theme

Supplementary Table 3: Expected Versus Prioritized EPMM Indicators by Country, Theme, and Stage of the Obstetric Transition

	Stage 2		Stage 3			Stage 4
**Indicator**	Cote d'Ivoire	Nigeria	Bangladesh	India	Pakistan	Mexico
1.1						
1.2						
1.3						
2.1	X					
2.2						
2.3						
3.1		X	X			
3.2			X			
3.3						
4.1		X			X	X
4.2						
4.3					X	
5.1	X		X			
5.2	X		X			
6.1		X			X	X
6.2					X	
6.3		X			X	X
7.1				X		
7.2						X
7.3						X
8.1				X		
8.2						
9.1						
9.2						
10.1	X					
10.2				X		
10.3	X		X	X	X	X
11.1						
11.2						
11.3						

Key

	Expected For Stage 2
	Expected for Stage 3
	Expected for Stage 4
X	Prioritized Theme

Note: Only expected indicators for themes that were prioritized by a given country are shaded.
